# Cell wall *O-*glycoproteins and *N-*glycoproteins: aspects of biosynthesis and function

**DOI:** 10.3389/fpls.2014.00499

**Published:** 2014-10-02

**Authors:** Eric Nguema-Ona, Maïté Vicré-Gibouin, Maxime Gotté, Barbara Plancot, Patrice Lerouge, Muriel Bardor, Azeddine Driouich

**Affiliations:** ^1^Laboratoire de Glycobiologie et Matrice Extracellulaire Végétale, UPRES EA 4358, Institut de Recherche et d’Innovation Biomédicale, Grand Réseau de Recherche-Végétal, Agronomie, Sol, Innovation, UFR des Sciences et Techniques, Normandie Université – Université de RouenMont-Saint-Aignan, France; ^2^Institut Universitaire de FranceParis, France; ^3^Plate-Forme de Recherche en Imagerie Cellulaire de Haute-Normandie, Institut de Recherche et d’Innovation Biomédicale, Faculté des Sciences et Techniques, Normandie UniversitéMont-Saint-Aignan, France

**Keywords:** arabinogalactan protein, cell wall, endoplasmic reticulum, extensin, glycan, glycosyltransferase, Golgi apparatus, plants

## Abstract

Cell wall *O-*glycoproteins and *N-*glycoproteins are two types of glycomolecules whose glycans are structurally complex. They are both assembled and modified within the endomembrane system, i.e., the endoplasmic reticulum (ER) and the Golgi apparatus, before their transport to their final locations within or outside the cell. In contrast to extensins (EXTs), the *O-*glycan chains of arabinogalactan proteins (AGPs) are highly heterogeneous consisting mostly of (i) a short oligo-arabinoside chain of three to four residues, and (ii) a larger β-1,3-linked galactan backbone with β-1,6-linked side chains containing galactose, arabinose and, often, fucose, rhamnose, or glucuronic acid. The fine structure of arabinogalactan chains varies between, and within plant species, and is important for the functional activities of the glycoproteins. With regards to *N-*glycans, ER-synthesizing events are highly conserved in all eukaryotes studied so far since they are essential for efficient protein folding. In contrast, evolutionary adaptation of *N-*glycan processing in the Golgi apparatus has given rise to a variety of organism-specific complex structures. Therefore, plant complex-type *N-*glycans contain specific glyco-epitopes such as core β,2-xylose, core α1,3-fucose residues, and Lewis^a^ substitutions on the terminal position of the antenna. Like *O-*glycans, *N-*glycans of proteins are essential for their stability and function. Mutants affected in the glycan metabolic pathways have provided valuable information on the role of *N-/O-*glycoproteins in the control of growth, morphogenesis and adaptation to biotic and abiotic stresses. With regards to *O-*glycoproteins, only EXTs and AGPs are considered herein. The biosynthesis of these glycoproteins and functional aspects are presented and discussed in this review.

## INTRODUCTION

Plants synthesize glycoconjugates that are structurally diverse and complex reflecting the diversity of plant physiological functions. The glycomolecules are usually assembled and modified within the plant endomembrane system, including the endoplasmic reticulum (ER), the Golgi apparatus and secretory vesicles responsible for their transport to different cell compartments/organelles including the cell wall. Their synthesis involves a number of steps, beginning with the formation of activated nucleotide sugars such as NDP-sugars or NMP-sugars (Bar-Peled and O’Neill, 2011). After their synthesis in the cytosol, the nucleotide sugars are then actively transported into the ER and Golgi stacks where they serve as donor substrates during glycan synthesis. Glycosyltransferases (GTs) transfer specific sugars from activated nucleotide sugars to a specific glycan acceptor leading to the extension of the glycomolecule involved. This occurs through a stepwise and sequential process which involves a number of different GTs of the secretory system. It is worth noting that 1.8% of *Arabidopsis thaliana*’s genome currently encode GT genes representing more than 462 GTs in total (Ulvskov et al., 2013).

Among the different plant organelles, the plant cell wall is a polysaccharide-rich extracellular compartment (Albersheim et al., 2011). In addition to polysaccharides, the plant cell wall also contains a significant percentage (∼10–15%) of *N*- and *O*-glycosylated proteins that are relatively less studied with regards to their biosynthesis and function. Both the *N*- and the *O-*glycosylation of proteins has a significant impact on both their structural properties and biological activities (Varki, 1993). Glycosylation and glycan processing are major *post-translational modifications* (PTMs) that cell wall proteins undergo inside the cell, and are considered important for their proper function. Indeed, in general, glycans are involved in the control of protein folding, cellular targeting and mobility, as well as signaling for regulation of plant growth, defense and different interactions with the surrounding environment (Varki and Lowe, 2009; Larkin and Imperiali, 2011; Cannesan et al., 2012; Nguema-Ona et al., 2013; Chen et al., 2014).

The *N-*/*O-* glycosylation of cell wall proteins is critical for plant development and responses to stress. Understanding and controlling *O-* and *N-*glycosylation of secreted proteins is also important in plant biotechnological applications.

## *N-*GLYCOSYLATED PROTEINS: SYNTHESIS AND FUNCTION

**FIGURE 1:**
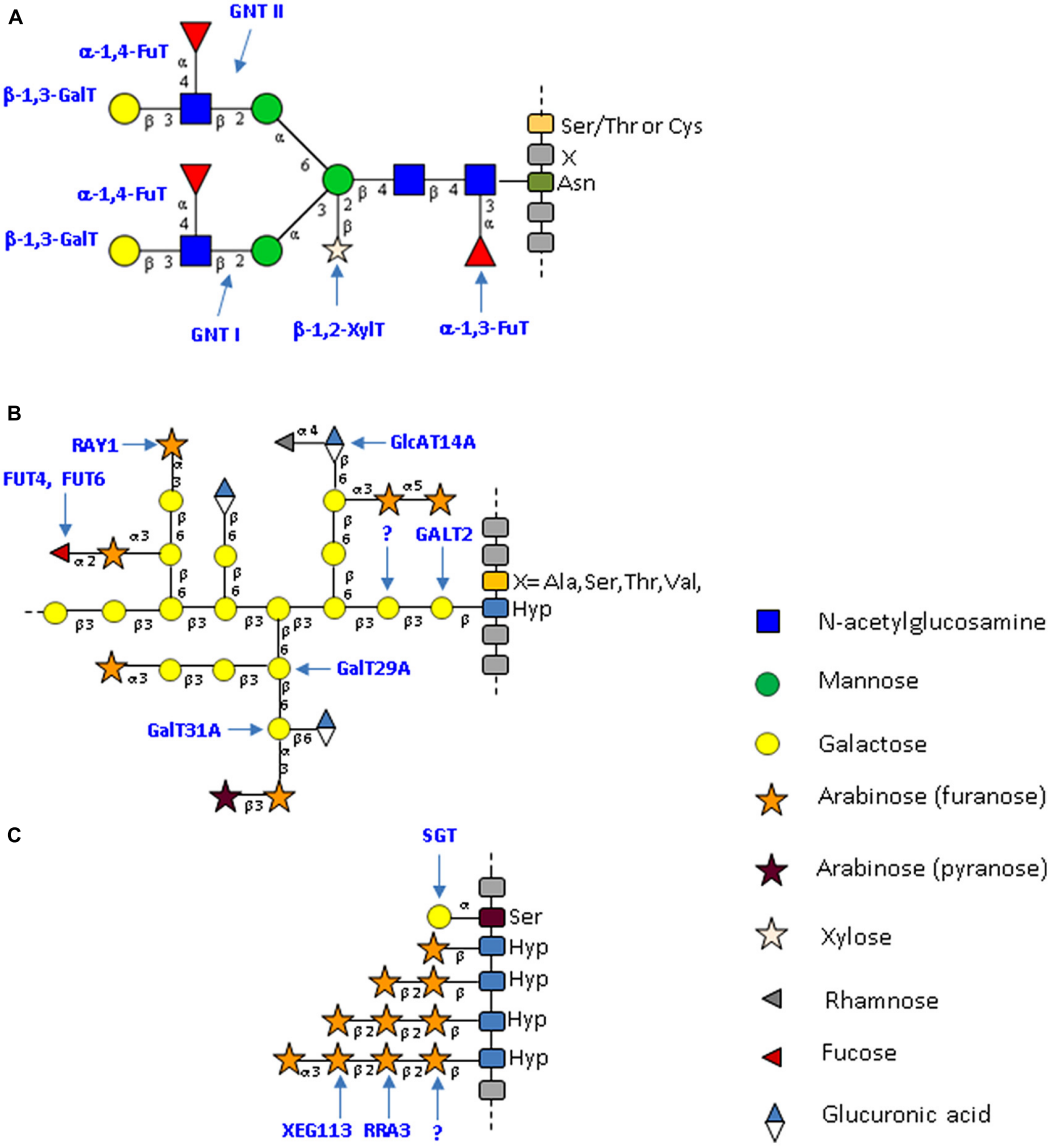
**Typical structure of plant *N*- and *O*-glycans from cell wall proteins. (A)** Specific complex-type *N*-glycans attached to plant glycoproteins. This *N*-glycan results from the action of a plant-specific repertoire of glycosyltransferases that lead to the formation of a glycan bearing plant-specific glyco-epitopes such as a core β-1,2-xylose; a core α-1,3-fucose and a Lewis^a^ antennae (Lerouge et al., 1998; Wilson et al., 2001b; Bardor et al., 2003, 2011). The *N*-glycan structures presented here are drawn according to the symbolic nomenclature adopted by the *Consortium for Functional Glycomics* (Varki et al., 2009). **(B)** Schematic representation of *O*-glycans (type II arabinogalactan) attached to AGPs. These glycans predominantly consist of arabinose and galactose. Minor sugars, such as glucuronic acid, fucose or rhamnose, are also present. The *O*-glycans are attached to non-contiguous Hyp residues. The model presented is modified from Tan et al. (2010) and Tryfona et al. (2012). **(C)** Schematic representation of *O*-glycans attached to plant EXT. These glycans consist of short chains of arabinose and on single galactose residues. The *O*-glycans are attached to contiguous Hyp residues. The model presented is modified from Saito et al. (2014). Yellow circle: galactose; green circle: mannose; blue square: *N*-acetylglucosamine; star: white xylose and red triangle: fucose; gray triangle: rhamnose; orange star: arabinose (furanose); purple star: arabinose (pyranose); blue/white diamond: glucuronic acid.

The *N*-glycosylation of proteins starts in the ER. ER-synthesizing events for *N*-glycans are highly conserved in all eukaryotes studied so far since they are instrumental for efficient protein folding (Aebi, 2013). The *N*-glycosylation pathway starts by the transfer *en bloc* of a lipid linked preassembled precursor (Glc_3_Man_9_GlcNAc_2_) by the oligosaccharyltransferase (OST) onto the *N*-glycosylation sites (Asn-X-Ser/Thr and/or Asn-X-Cys) of the nascent proteins (Burda and Aebi, 1999; Gil et al., 2009; Zielinska et al., 2010; Matsui et al., 2011). The α-glucosidases I and II then remove two glucose residues from the *N-*glycan resulting in the presence of only one terminal glucose on the glycoprotein. This allows its entry into the ER control quality cycle (Aebi, 2013). Once the glycoprotein is correctly folded, the last glucose residue is removed by the α-glucosidase II prior to its transport into the Golgi apparatus where further modifications occur including removal of mannose residues and sequential addition of specific sugars through the action of GTs resulting in the formation of complex-type *N-*glycans. In plants, many genes encoding for Golgi GTs have already been identified (**Table 1**). These include, for example, *N-*acetylglucosaminyltransferase I (GnT I; Bakker et al., 1999; Strasser et al., 1999a; Wenderoth and von Schaewen, 2000), *N-*acetylglucosaminyltransferase II (GnT II; Strasser et al., 1999b), core α-1,3-fucosyltransferase (α1,3-FuT; Leiter et al., 1999; Wilson et al., 2001a), β-1,2-xylosyltransferase (β1,2-XylT; Strasser et al., 2000; Pagny et al., 2003), Lewis-type α-1,4-fucosyltransferase (α1,4-FuT Bakker et al., 2001; Wilson et al., 2001b; Léonard et al., 2002), and β-1,3-galactosyltransferase (β1,3-GalT; Strasser et al., 2007). In contrast to the ER steps, evolutionary adaptation of *N-*glycan processing in the Golgi apparatus has given rise to a variety of organism-specific complex structures (Varki, 2011). Therefore, more complex plant *N-*glycans consist of specific glyco-epitopes such as core β-1, 2-xylose, core α-1,3-fucose residues, and Lewis^a^ substitutions on the terminal position of the antenna (**Figure 1A**; Lerouge et al., 1998; Bardor et al., 2003, 2011; Strasser et al., 2004).When abnormally processed, *N-*glycosylated proteins cause major developmental disorders and are usually associated to diseases in mammals (Ioffe and Stanley, 1994; Metzler et al., 1994; Lowe and Marth, 2003; Hennet, 2012). In plants, abnormal *N-*glycosylated proteins rarely present developmental disorders under normal growth conditions (von Schaewen et al., 1993; Strasser et al., 2004). However, the cellulose-deficient *Arabidopsis* mutant *rsw3* which is defective in the catalytic subunit of the α-glucosidase II presents radially swollen roots and a deficiency in cellulose content (Burn et al., 2002). Moreover, under stress conditions (e.g., salt), modified phenotypes such as abnormal plant growth (Strasser et al., 2007) or altered root growth in the *Arabidopsis cgl* mutants (Kang et al., 2008; von Schaewen et al., 2008), have been observed. Indeed, in these studies, reduced root growth and abnormal root morphology were observed for *Arabidopsis* plants cultivated on media containing high NaCl concentration. In contrast to *Arabidopsis*, a severe phenotype with arrested seedling development and premature death before reaching the reproductive stage has been reported recently for rice *gntI* mutant (Fanata et al., 2013). Such plants also present defects in cell wall composition, especially reduced cell wall thickness, and decreased in cellulose content as well as reduced sensitivity to cytokinin. Plant complex-type *N-*glycans are ascribed to many biological functions in relation with plant development that have been recently reviewed by Strasser (2014; this issue). These include effects on plant innate immunity, tolerance to abiotic stress and root development. Therefore these functional aspects will not be further described in this review.

**Table 1:** Known enzymes involved in plant *N*- glycans and *O*- cell wall glycan biosynthesis.

AGP glycan biosynthetic enzymes	CAZy family	Protein name	Origin	Reference
Hydroxyproline *O*-galactosyltransferase	GT31	*At*GALT2	*Arabidopsis thaliana*	Basu et al. (2013)
β-1,3-galactosyltransferase	GT31	*At*1g77810	*Arabidopsis thaliana*	Qu et al. (2008)
β-1,6-galactosyltransferase	GT31	*At*GalT31A	*Arabidopsis thaliana*	Geshi et al. (2013)
–	GT29	*At*GalT29A	*Arabidopsis thaliana*	Dilokpimol et al. (2014)
Arabinofuranosyltransferase	GT77	RAY1	*Arabidopsis thaliana*	Gille et al. (2013)
β-glucuronosyltransferase	GT14	*At*GlcAT14A	*Arabidopsis thaliana*	Knoch et al. (2013)
α-1,2-fucosyltransferase	GT37	*At*FUT4	*Arabidopsis thaliana*	Wu et al. (2010)
–	GT37	*At*FUT6	*Arabidopsis thaliana*	Wu et al. (2010)
**Extensin glycan biosynthetic enzymes**	**CAZy family**	**Protein name**	**Origin**	**Reference**
Serine *O*-galactosyltransferase	unknown	SGT1	*Chlamydomonas reinhardtii; Arabidopsis thaliana*	Saito et al. (2014)
Arabinosyltransferase	GT77	RRA3	*Arabidopsis thaliana*	Velasquez et al. (2011)
–	GT77	XEG113	*Arabidopsis thaliana*	Gille et al. (2009)
***N*-glycan biosynthetic enzymes**	**CAZy family**	**Protein name**	**Origin**	**Reference**
Oligosaccharyltransferase		OST	*Arabidopsis thaliana; Oryza sativa*	Farid et al. (2013), Qin et al. (2013)
α-glucosidase I		GCS I	*Arabidopsis thaliana*	Boisson et al. (2001)
α-glucosidase II		GCS II	*Solanum tuberosum*	Taylor et al. (2000)
α-mannosidase I		MNS 1-3	*Arabidopsis thaliana*	Liebminger et al. (2009)
*N*-acetylglucosaminyltransferase I	GT13	GnT I	*Arabidopsis thaliana; Nicotiana tabacum; Solanum tuberosum*	Bakker et al. (1999), Strasser et al. (1999a), Wenderoth and von Schaewen (2000)
α-mannosidase II		GM II	*Arabidopsis thaliana*	Strasser et al. (2006)
*N*-acetylglucosaminyltransferase II	GT16	GnT II	*Arabidopsis thaliana*	Strasser et al. (1999b)
α-1,3 fucosyltransferase	GT10	α-1,3-FuT	*Vigna radiata; Arabidopsis thaliana; Medicago sativa*	Leiter et al. (1999), Wilson et al. (2001a), Sourrouille et al. (2008)
β-1,2-xylosyltransferase	GT61	β-1,2-XylT	*Arabidopsis thaliana*	Strasser et al. (2000), Pagny et al. (2003), Bencúr et al. (2005)
β-1,3-galactosyltransferase	GT31	β-1,3-GalT	*Arabidopsis thaliana*	Strasser et al. (2007)

## *O-*GLYCOSYLATED CELL WALL PROTEINS, ARABINOGALACTAN PROTEINS, AND EXTENSINS

Plant *O*-glycosylated cell wall proteins belong to the superfamily of hydroxyproline-rich glycoproteins (HRGPs). This superfamily of plant cell wall proteins which account for nearly 10% of the dry weight of the wall, is characterized by a high proline (Pro) content. Furthermore many of these Pro residues become hydroxylated (hydroxyproline, Hyp) during synthesis and consequently become glycosylated in various ways. Pro residues are distributed at different sites within the sequence and these patterns have suggested different classifications of HRGP members into different groups. EXTs and arabinogalactan proteins (AGPs) are two *O-*glycosylated HRGP subfamilies which have gained much attention (Kieliszewski and Shpak, 2001; Showalter, 2001; Schultz et al., 2002; Showalter et al., 2010; Kieliszewski et al., 2011; Lamport et al., 2011; Nguema-Ona et al., 2012, 2013; Tan et al., 2012; Velasquez et al., 2012). The nature of sugars being incorporated and the level of glycosylation vary between these two families, but also within the members of these subfamilies. For example, Kieliszewski et al. (2011) have shown that occurrence of contigs of 3–5 Hyp, preceded by a serine residue (Ser-Hyp_4_) led to the synthesis of a short arabinoside of 3–5 residues. Serine residue in the Ser-Hyp_4_ contig is often *O-*glycosylated with a single galactose (Velasquez et al., 2012; Saito et al., 2014). This action is performed by serine-*O*-galactosyltransferases (Ser-*O*-Gal-T), specific to plants (Saito et al., 2014). Non-contiguous Hyp residues rather lead to the synthesis of a large arabino-galactosylated glyco-epitope on the protein (Kieliszewski and Lamport, 1994; Shpak et al., 1999; Kieliszewski et al., 2011).

Arabinogalactan proteins and EXTs have been studied for decades, and shown to fulfill many functions related to development, and responses to biotic and abiotic stresses in plants (Hall and Cannon, 2002; Motose et al., 2004; Lee et al., 2005; Nguema-Ona et al., 2007, 2013; Seifert and Roberts, 2007; Cannon et al., 2008; Ellis et al., 2010; Lamport et al., 2011; Velasquez et al., 2011; Cannesan et al., 2012; Moore et al., 2014a,b). These studies have emphasized the importance of their *O-*glycan structures. Indeed, AGPs and EXTs are decorated with complex to simple carbohydrate-chains () that are required for functionality of these glycomolecules. Until recently, the enzymes, as well as the molecular mechanisms controlling the synthesis of HRGP *O-*glycans, were poorly understood. A recent effort in the identification of the genes involved in the biosynthesis of HRGP *O-*glycans has considerably improved our understanding of the molecular events controlling the addition of sugars on these Hyp-rich proteins. The aim of this section is to bring together recent advances in the biosynthesis of HRGP *O-*glycans, with a focus on AGPs and EXTs. Structural and biological functions are also discussed.

## AGPs AND EXTs: THE SYNTHESIS OF *O*-GLYCANS

### PRO HYDROXYLATION OF HRGPs

Pro hydroxylation of plant cell wall HRGPs occurs predominantly on Hyp that are formed in the secretory pathway through the action of proline hydroxylases (P4Hs). In *Arabidopsis*, 13 P4Hs have been identified (Hieta and Myllyharju, 2002; Vlad et al., 2007; Velasquez et al., 2011). P4Hs are membrane-anchored enzymes (Yuasa et al., 2005). It is likely that Pro hydroxylation begins in the ER and continues in the Golgi apparatus. Detailed investigations of substrate affinity of two *Arabidopsis* P4-Hs, *At*P4H1 and *At*P4H2, showed that both *At*P4H1 and *At*P4H2 hydroxylate AGP-like and EXT-like synthetic peptides (Hieta and Myllyharju, 2002; Tiainen et al., 2005). However, the substrate specificity of the enzymes towards the two classes of synthetic peptides differed. Additional data showed that *At*P4H2 poorly hydroxylated animal collagen, but did not hydroxylate animal hypoxia-inducible transcription factor (HIF); while *At*P4H1 hydroxylates both animal Hyp-containing proteins collagen and HIF (Hieta and Myllyharju, 2002). Similarly, Velasquez et al. (2011) showed that some root hair-specific P4Hs are able to hydroxylate EXTs, and displayed almost no activity toward AGP-like peptides. Root hair morphology of *Arabidopsis p4h* mutants was dramatically altered. Complementation of these mutants with wild type genes restored the phenotype. In addition to being substrate-specific, Velasquez et al. (2011) also showed that some P4Hs were also cell type-specific: P4H2 and P4H5 being confined to trichoblast cells, while P4H13 being present in both trichoblast and atrichoblast cells. Recently, it has also been shown that different tomato P4Hs played a role in plant growth, and exhibited substrate- and tissue-specific activities (Fragkostefanakis et al., 2014). The authors have shown that silencing individual P4Hs result on an increased expansion of root and leaf cells in tomato. This increase correlated with a reduction in the amount of AGPs and possibly EXTs. Plants are therefore likely to regulate the secretion of various classes of HRGPs at different stages of development and/or responses to stress to perform specific functions in a given cell type or organ. After their synthesis and secretion, HRGPs may be modified and/or re-arranged in the cell wall, but this aspect has received little attention so far, particularly in the case of AGPs.

### GLYCOSYLTRANSFERASES INVOLVED IN AGP *O*-GLYCAN BIOSYNTHESIS

Liang et al. (2010) has suggested that ∼15 GTs are involved in AGP glycan biosynthesis. Initiation of the biosynthesis requires the action of specific AGP *O*-Hyp Gal-T, able to initiate the galactosylation of hydroxylated residues on AGP backbone. Recently, an *Arabidopsis* Gal-T (*At*GalT2), belonging to CAZy GT family 31 and containing a pfam 01762 domain encoding a Gal-T catalytic domain, able to add one galactosyl residue to Hyp residues of synthetic AGP-like peptides, has been identified (Basu et al., 2013). The authors showed that *At*GalT2 was able to add one galactose residue to synthetic AGP-like peptide, and not to synthetic EXT-like peptides. *At*GalT2 was also harboring a GAL-LECTIN binding domain pfam 00337. This domain, previously identified as a *N*-acetylgalactosaminyl GT (CAZy GT family 27), was involved in catalyzing the first steps of the glycosylation of mammalian mucins (Hassan et al., 2000; Wandall et al., 2007). *At*GAlT2 was found to be located in the ER and in the Golgi apparatus, a pattern similar to the one displayed by P4Hs (Yuasa et al., 2005; Velasquez et al., 2011). It is possible that these two enzymes co-operate in plants to hydroxylate Pro residues and add the first galactosyl residue of the newly synthesized β-1,3- galactan chain. In addition to *At*GalT2, Qu et al. (2008), using a combination of bioinformatic approaches, identified several additional Gal-Ts belonging to the GT family 31, and showed their putative involvement in the elongation of the β-1,3- galactan backbone of AGPs. For instance, the protein encoded by the gene *At*1g77810 was demonstrated to exhibit a specific β-1,3-Gal-T activity. An additional Gal-T activity that adds the second galactose to the Gal-Hyp nascent chains has also been partially characterized (Liang et al., 2010).

In addition to β-1,3-Gal-T, AGP glycan synthesis also requires the action of different other β-1,6-Gal-T, α-1,3- and α-1,5-arabinosyltransferase (Ara-T), β-glucuronosyltransferase (GlcA-T), and α-1,2-fucosyltransferase (FuT; Wu et al., 2010). Recently, two *Arabidopsis* Gal-Ts showing a β-1,6-Gal-T activity have been identified: *At*GalT31A, a β-1,6-Gal-T which is classified into the CAZy GT family 31, is required for the addition of Gal residues to existing β-1,6- galactan chains (Geshi et al., 2013) while *At*GalT29A (CAZy GT family 29) is required for the addition of galactose residues to β-1,3- and β-1,6- galactan chains (Dilokpimol et al., 2014). Both *At*GalT31A and *At*GalT29A are type II transmembrane proteins located in the Golgi apparatus. Traces of ER-localization previously observed with P4Hs and *At*GalT2 were not found, suggesting that addition of β-1,6-galactose residues to the side chains of AGPs occurs later during the transit of nascent HRGPs into Golgi stacks. Using subcellular co-localization approaches, FRET acceptor photo-bleaching techniques as well as immuno-precipitation techniques, the authors showed that (i) *At*Galt31A and *At*Gal29A were organized into heterodimer complexes, and (ii) this heterodimer had an enhanced enzymatic activity than the homodimer *At*GalT31A/*At*GalT31A, or *At*GalT29A/*At*GalT29A. AGP arabinogalactan chains are also modified with glucuronic acid (GlcA) residues. Knoch et al. (2013) have identified an *Arabidopsis* transferase belonging to the CAZy GT family 14, named *At*GlcAT14A, exhibiting an AGP-specific GlcA-T activity, able to transfer GlcA residues both onto β-1,3- and β-1,6-galactan chains (see also Zhou et al., 2009; Ye et al., 2011). Interestingly, *At*GlcAT14A was localized to the Golgi apparatus. *At*GlcAT14A is co-expressed with *At*GalT31A and co-localize in the Golgi apparatus. However, the FRET photo-bleaching acceptor technique showed that both enzymes did not physically interact. These findings suggest that all the enzymes involved in AGP glycan synthesis, although probably co-regulated, are not necessarily part of a unique multi-protein complex. *Arabidopsis* AGP glycans were also shown to contain fucose residues (Tryfona et al., 2012). Two *Arabidopsis* FuT *At*FUT4 and *At*FUT6, belonging to the CAZy GT family 37, were shown to specifically add fucose residues to tobacco arabinogalactosylated AGP glycan chains (Wu et al., 2010). Interestingly, de-arabinosylation of tobacco AGP glycans (using arabinofuranosidase) prevented the addition of fucose residue to the glycan, suggesting that arabinosylation was required for further addition of fucose by *At*FUT4 and *At*FUT6, supporting the arguments for sequential synthesis of AGP glycans along the Golgi cisternae. Biochemical data showed that both FuTs fucosylate AGP glycan in a different manner, most likely on different arabinose residues (Wu et al., 2010). Finally, arabinose, along with galactose, is the more abundant sugar found in AGP glycans. Recently, Gille et al. (2013) have identified an AGP-altered mutant of *Arabidopsis* named *reduced arabinose yariv1* (*ray1-1*). Monosaccharide composition of a root AGP fraction precipitated with β-glucosyl Yariv, showed a significant decrease in arabinose content in the *ray1-1* mutant, as compared to the wild type. In addition, the *ray1-1* mutant showed a reduction in the length of its primary roots. *RAY1-1* gene was found to encode for a CAZy GT family 77 Ara-T, localized in the Golgi apparatus (Gille et al., 2013). It is however, unknown if RAY1 is able to add arabinosyl residues to short oligo-arabinosides also found on AGPs.

### EXT *O*-GLYCAN BIOSYNTHESIS

Extensin *O-*glycans consists of short arabinoside chains with single galactose residues, linked respectively to Hyp residues, and serine residues of the Ser-Hyp_4_ motifs. In contrast to the length and the molecular weight of arabinogalactan chains found in AGPs; EXT arabinoside chains are limited to 4–5 arabinosyl residues, predominantly β-1,2-linked. The number of enzymes required for their biosynthesis is also reduced to Ara-T initiating and elongating the arabinoside chains, and to the Ser-*O*-Gal-T, adding the single galactose residue to serine (Velasquez et al., 2012; Saito et al., 2014). Ser-*O*-Gal-T are type I transmembrane proteins, located in the ER and possibly in the *cis*-Golgi cisternae (Saito et al., 2014), and prior hydroxylation of Pro residues is required for galactosylation of serine residues on EXTs. It is unknown if this initial galactosylation is required for further EXT arabinosylation.

While the enzyme adding the first galactose residue to AGPs is now identified, the enzyme transferring the first arabinosyl-residue to *O-*Hyp EXT (and maybe on Ser-Hyp_3_ domains of certain AGPs; Qi et al., 1991), remains unidentified. However, Ara-Ts adding the second, the third, and then the fourth arabinose residue to *O-*Hyp EXTs have been identified. Indeed, *Arabidopsis* RRA1-3, XEG113, and ExAD were shown (or proposed for ExAD; Velasquez et al., 2012) to transfer respectively, the second, the third and the fourth arabinose residue β-1,2-linked to EXT (Egelund et al., 2007; Gille et al., 2009; Velasquez et al., 2011, 2012). XEG113 belongs to the CAZy GT family 77 and *xeg113* mutants exhibited abnormally elongated hypocotyls under stress conditions. XEG113 was found to be associated with Golgi membranes (Gille et al., 2009). RRA3 is also a type II transmembrane protein, member of the CAZy GT family 77, localized in the Golgi apparatus, and shown to reduce root hair growth. *Rra3 Arabidopsis* mutants exhibited impaired root hairs. Using a base-mediated hydrolysis of the peptide backbone, followed by mass spectrometry analyses, the authors elegantly showed that XEG113 was responsible for the addition of the second arabinose residue to an elongating β-1,2- arabinan chain, while RRA3 was responsible for the addition of the third residue. RRA3, XEG113, P4H2, and P4H5 were co-expressed, also indicating that EXT glycan synthesis must be tightly regulated within the endomembrane system. Moreover, different cell wall related proteins including *At*RSH1 (a classical EXT HRGP), *At*LRX1 (a hybrid EXT HRGP), *At*PRP1 (a proline-rich protein), and several peroxidase genes were also co-expressed with Ara-T and P4Hs, in *Arabidopsis* root hairs (Velasquez et al., 2011).

Together, these studies suggest that AGP and EXT glycan synthesis is initiated in the ER and continues in the Golgi apparatus, similarly to the *N-*glycosylation pathway. P4Hs and *At*GalT2 may co-operate during hydroxylation and galactosylation of AGP in the ER and in the Golgi apparatus. Elongation and ramification of the AGP glycans would probably take place in different Golgi subcompartments before their export to the cell surface. But specific compartmentalization of the enzymes involved in HRGPs synthesis within specific Golgi cisternae is not yet established and requires further investigations. Such an arrangement has already been described for enzymes involved in the *N-*glycosylation of secreted proteins (Saint-Jore-Dupas et al., 2006) and for the synthesis of the hemicellulosic polysaccharide xyloglucan (Chevalier et al., 2010; Driouich et al., 2012).

## AGPs AND EXTs: ROLE IN MORPHOLOGY AND DEVELOPMENT

Cell wall components are organized into networks of polysaccharides and glycoproteins which, apart from operating individually, are strongly interconnected (Carpita and Gibeaut, 1993; Burton et al., 2010; Albersheim et al., 2011). Indeed, it is widely acknowledged that cellulose microfibrils and hemicellulose constitute a primary network of polysaccharides, embedded into a second network made of pectic polysaccharides. Less often referred as such, *O-*Hyp cell wall proteins constitute the third network of the wall component. Bridges between these three networks do also exist and structural alteration occurring on a single cell wall component often affects overall cell wall architecture and integrity. Thus, structurally altered AGPs/EXTs weaken cell wall architecture (both covalently and non-covalently), and affect biological processes controlled by the cell wall compartment.

Indeed, most of the *Arabidopsis* mutants defective in one or more enzymes described above presented various developmental and morphological alterations. van Hengel and Roberts (2002) showed that the lack of fucose residue in the *Arabidopsis mur1* mutant caused their roots to be shortened. This growth defect was due to structural modification of root AGPs (van Hengel and Roberts, 2002), as well as a result of altered rhamnogalacturonan-II synthesis, since the disorder was partially rescued by exogenous application of boric acid (O’Neill et al., 2001). Liang et al. (2013) showed that a deficiency in the genes *AtFUT4* and *AtFUT6* caused a reduction of root growth under saline stress conditions (see also Tryfona et al., 2014). The lack of fucose residue was proposed to affect intramolecular interactions between AGPs and other wall components. Similarly reduced galactosylation of AGPs in *reb1-1* mutant of *Arabidopsis* caused strong swelling of trichoblast cells as well as reduced root growth (Andème-Onzighi et al., 2002; Nguema-Ona et al., 2006). The *Arabidopsis* mutant *atglcat14a*, deficient in an AGP-specific GlcA GT, showed an abnormal increase in root and hypocotyl length, when compared to the wild type (Knoch et al., 2013). Biochemical analysis in this mutant showed an alteration in the AGP composition and associated glycosidic linkages as compared to the wild type, suggesting that biochemical phenotype indirectly impacts cell elongation *via* an overall change in cell wall architecture and integrity. The mutation in *At*GalT31A caused the arrest of the embryo development at the globular stage, while complementation of the mutant with *At*GALT31A restored the wild type phenotype, thus linking the requirement of correctly glycosylated AGPs with the progression of embryogenesis beyond the globular stage (Geshi et al., 2013). However, a study of *atgalt2* deficient *Arabidopsis* mutants showed that allelic mutant lines contained less Gal-T activity when compared to the wild type without displaying any significant alteration of the phenotype. Basu et al. (2013) suggested that other Hyp*-O* Gal-Ts may compensate for the loss of *At*GalT2, and that examination of these mutants under non-physiological conditions, or the production of multigene mutants within this gene family may reveal novel phenotypes. Recently, an unusual AGP (named APAP1) was found to be covalently linked to pectin rhamnogalacturonan-I and to arabinoxylans (Tan et al., 2013). Absence of APAP1 in the corresponding mutant led to an increased extractability of pectins and xylans, thus suggesting the alteration of its overall wall architecture. *Apap1* mutants exhibited a significant increase in the height inflorescence stem, although the overall morphology was comparable to that of the wild type.

*Arabidopsis* EXT-deficient or EXT-altered mutants also presented various developmental and morphological alterations, due to an alteration in their overall wall architecture. Velasquez et al. (2011) showed that disrupting the Pro hydroxylation and/or improper *O-*glycosylation impacted EXT ability to form covalent intra and inter-molecular network in the wall. Indeed, secondary helix conformation found in EXTs, required for normal catalysis of the di-isodityrosine bondages by wall peroxidases (Held et al., 2004), was altered in P4H-deficient and Ara-T-defective *Arabidopsis* mutants. The authors concluded that the absence, or the alteration of their Hyp-*O-*arabinosides, destabilized the EXT helical secondary structure, altering their ability to interact in the wall with other cell wall components, thus altering their structural function *in muro*. Interestingly, unlike in plants, the hydroxylated Pro residues of animal proteins are not glycosylated. Pro hydroxylation itself is sufficient for the conformational stability of animal Hyp-rich proteins such as collagen. This PTM is generally sufficient for proper functioning of such proteins. Indeed, Hyp stabilizes their triple helical structure at body temperature (Kivirikko and Pihlajaniemi, 1998; Myllyharju, 2003), and to date, no animal Hyp-containing proteins have been found to be glycosylated. *O-*glycosylation of Hyp is a rather plant-specific PTM, required for the proper functioning of plant Hyp-containing proteins including HRGPs. While the enzymes hydroxylating Pro residues on AGPs and EXTs are similar to mammalian systems, the ones that initiate and elongate the glycan chains of these HRGPs are unique to plants.

Furthermore, the study of the *Arabidopsis rsh* mutant, deficient in an EXT (RSH/EXT3) has also shown the importance of EXTs for normal plant cell wall architecture and function in development. RSH/EXT3 is an *Arabidopsis* EXT which was shown to play a key role during cytokinesis, by controlling cell plate formation (Hall and Cannon, 2002; Cannon et al., 2008). RSH/EXT3 positively charged was proposed to interact with negatively charged pectins to create a template for newly synthesized cell walls.

## AGPs AND EXTs: ROLE IN BIOTIC STRESS

**FIGURE 2:**
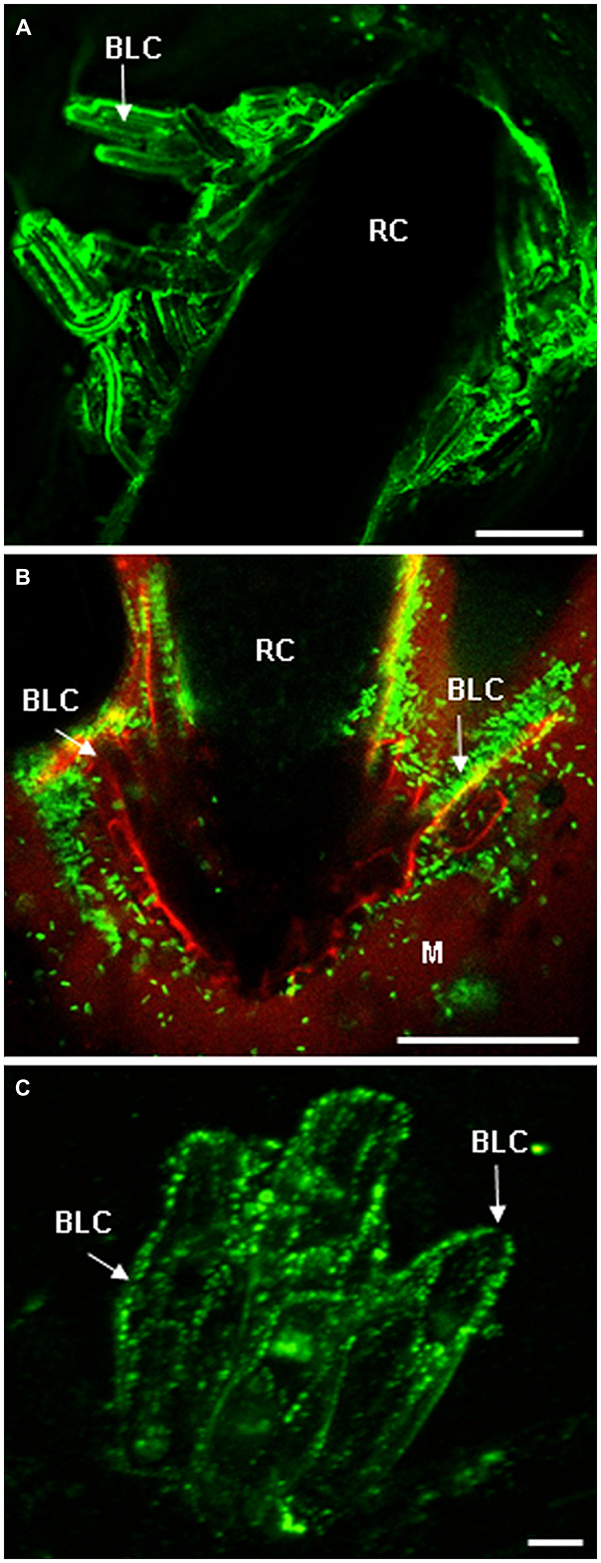
**Root cap (RC) and border cells are both enriched in AGP and EXT epitopes. (A)** Immunostaining of AGP epitopes at the surface of RC and border-like cells of *Brassica napus* with the mAb JIM8 (from Cannesan et al., 2012 with permission). Root border-like cells are produced and released from the RC. **(B)** Micrographs showing the association between root border-like cells from *Arabidopsis thaliana* and *Rhizobium* sp. YAS34-GFP. The GFP-expressing bacteria appear green at the root surface (from Vicré et al., 2005 with permission). This association is AGP-dependant as demonstrated in Vicré et al. (2005). **(C)** Fluorescent micrographs of root border-like cells from flax (*Linum usitatissimum*) immunostained with the monoclonal antibody LM1 specific for EXT epitopes (from Plancot et al., 2013 with permission). Bars = 20 μm **(A)**, 50 μm **(B)**, and 8 μm **(C)**. BLCs, border-like cells; M, mucilage; RC, root cap.

In addition to their role in morphology and growth, AGPs and EXTs were shown to play key roles in plant responses to biotic stress. Esquerré-Tugayé (1979) and Esquerré-Tugayé et al. (1979) have shown that plants respond to fungal infection by an increased secretion of HRGPs. Both AGPs (reviewed in Nguema-Ona et al., 2013) and EXTs were later on shown to play various roles in this response to pathogens. More specifically root apices and exudates were found to be enriched in AGPs (**Figure 2**), their chemical composition being different depending on both root tissues and plant species (Dolan et al., 1995; Durand et al., 2009; Cannesan et al., 2012). AGPs have long been suspected to be involved in root-microorganisms interaction including symbiotic associations (Scheres et al., 1990; Balestrini et al., 1996; Berry et al., 2002). For instance, alteration of AGP synthesis or secretion was shown to inhibit *Rhizobium* sp. YAS34 attachment to the root surface of *Arabidopsis thaliana* (Vicré et al., 2005; **Figure 2**). Xie et al. (2012) further demonstrated that AGPs from pea root exudates promote polar orientation and adhesion of *Rhizobium leguminosarum*. However, AGP functioning in root defense remained speculative until recently, as demonstrated by the study of Cannesan et al. (2012) on pea roots. The authors have shown that AGPs isolated from root cap (RC) and border cells are strong attractants of zoospores of the pathogenic oomycete *Aphanomyces euteiches in vitro* (Cannesan et al., 2012). The AGPs also inhibited *in vitro* cyst germination and the subsequent mycelium growth and propagation. These findings highlight the important contribution of AGPs in *Aphanomyces euteiches* root infection and show for the first time that AGPs are involved in controlling root-pathogenic oomycete interaction (see also Nguema-Ona et al., 2013).

Extensins have also been shown to play a significant role in plant defense and protection against bioagressors. Immunolocalization studies using the mAbs JIM 20 and JIM 11 revealed the abundant presence of EXT epitopes in cell walls of the resistant wax gourd cultivar to *Fusarium oxysporum* as compared to susceptible cultivar (Xie et al., 2011). In addition, elicitation with fusaric acid or infection with *F. oxysporum* caused important decrease of the immunofluorescence in both resistant and susceptible cultivars. Also, elicitation of grapevine callus cultures resulted in both the insolubilization of a specific 89.9 kD EXT and the induction of the catalytic activity of an EXT peroxidase (Jackson et al., 2001). Furthermore, EXTs have been shown to accumulate in response to the pathogenic oomycete *Sclerospora graminicola* in resistant pearl millet cultivar (Deepak et al., 2007). The high content of EXTs was tightly correlated with an increase in the levels of isodityrosine and H_2_O_2_ suggesting cell wall strengthening in the resistant cultivar presumably to limit *Sclerospora graminicola* penetration and tissue infection.

More recently, the implication of EXTs as part of the innate immune response of root border-like cells (BLCs) of *Arabidopsis thaliana* and *Linum usitatissimum* has been investigated by Plancot et al. (2013). Root border cells from plants such as pea, soybean, or cotton are highly specialized in root protection and production of various anti-microbial compounds (Hawes et al., 2000, 2003). Although such a function still needs to be clearly established for BLCs, a class of border cells that is relatively less studied. Recent work suggests a role for these BLCs in root defense (Driouich et al., 2013). Plancot et al. (2013) have also demonstrated that, in response to elicitors (e.g., flagellin 22), a significant increase in the production of H_2_O_2_ was detected in root BLCs together with a strong activation of genes involved in EXT biosynthesis and cross-linking. This is consistent with the finding of Velasquez et al. (2011) which showed that EXTs biosynthesis genes were co-expressed with peroxidase genes. Interestingly, treatment with elicitors also caused modifications in the distribution of EXT epitopes within cell walls of root BLCs (**Figure 2**). The effect of elicitation on the pattern of labeling with the mAb LM1 was shown to depend on both the nature of elicitors and plant species. Elicitation with flagellin 22 almost abolished immunostaining of LM1-recognized epitopes reflecting reorganization of the EXT network within the cell wall due to extensive cross-linking. Such an oxidative cross-linking of EXTs may result in a reinforced glyco-network that enhances physical properties of the cell wall in both *Arabidopsis thaliana* and *L. usitatissimum* (Plancot et al., 2013). This reinforcement of the cell wall would in turn limit/prevent penetration and progression of pathogens within root tissues.

Together these findings strongly suggest that AGPs and EXTs are key components of root protection, and more specifically of root border cells. However, further investigations where root border cells are directly challenged with specific pathogens are needed to provide a biological context for these observations. So far, the immune response in roots remains poorly understood and appears to be highly complex and cell-type specific (Millet et al., 2010; Cannesan et al., 2012; Balmer and Mauch-Mani, 2013). To our knowledge, the only study that clearly demonstrated the relationship between the production of EXT and plant resistance to pathogens was performed in leaf tissues (Wei and Shirsat, 2006). In this study, over-expression of the *EXT1* gene in leaves of *Arabidopsis thaliana* clearly limits the spreading of the pathogenic bacteria *Pseudomonas syringae* DC3000 within the tissues. Subsequently, the infection symptoms are significantly reduced. It is clear that the implication of EXT and AGP populations in root protection is far from being fully understood and more studies are needed to elucidate the role of individual HRGPs/or their glycans in resistance to biotic stress.

## CONCLUSION AND OUTLOOK

Like *N-*glycoproteins, cell wall *O-*glycoproteins, AGPs and EXTs, are synthesized, assembled and modified within the secretory system. Their glycans, although structurally different and diverse, play a major role in their stability, activity and function. Both types of glycoproteins were shown to be involved in the control of many biological activities and physiological processes in various plant species. However, the specific role of each glycan type and the associated oligosaccharides in biological processes is not known. One of the important challenges for the future is to elucidate the contribution of each of these glycans (and associated sugars) in regulating cell growth, development and adaptation of plants to environmental stresses, either biotic or abiotic. Even more challenging is the search for potential relationships between a given glycan/oligosaccharide structure and a given function in a given tissue. For instance, how specific *O-*glycan structures regulate morphology, growth or biotic interactions of certain root cell types with microbes is a major issue that deserves further attention.

Recently, a number of the carbohydrate active enzymes involved in *N-* and *O-*glycan metabolism have been identified and have advanced our understanding of the biosynthetic machineries of these glycoproteins. How these enzymes are spatially organized and assembled within different compartments of the endomembrane system (i.e., specifically within Golgi subcompartments and Golgi-derived secretory vesicles) and how these are regulated during development is not fully understood and remains an exciting research opportunity for the future.

## Conflict of Interest Statement

The authors declare that the research was conducted in the absence of any commercial or financial relationships that could be construed as a potential conflict of interest.
